# Genome analysis of *Salmonella enterica* serovar enteritis strains isolated from poultry and humans in Burkina Faso

**DOI:** 10.1128/mra.01024-23

**Published:** 2024-05-03

**Authors:** Assèta Kagambèga, Hazem Ramadan, Michel Dione, Soutongnooma C. Bouda, Lari M. Hiott, Elizabeth A. McMillan, Poonam Sharma, Sushim K. Gupta, Nicolas Barro, Charlene R. Jackson, Jonathan G. Frye

**Affiliations:** 1 United States Department of Agriculture, Poultry Microbiological Safety and Processing Research Unit, U.S. National Poultry Research Center, Agricultural Research Service, Athens, Georgia, USA; 2 Laboratoire de Biologie Moléculaire, D'épidémiologie et de Surveillance des Bactéries et Virus Transmissibles par les Aliments (LaBESTA)/Ecole Doctorale Sciences et Technologies (EDST)/Université Joseph KI-ZERBO, Ouagadougou, Burkina Faso, Burkina Faso; 3 Ecole Normale Supérieure, Ministère des Enseignement Supérieur, de la Recherche Scientifique et de L'innovation, Ouagadougou, Burkina Faso, Burkina Faso; 4 Hygiene and Zoonoses Department, Faculty of Veterinary Medicine, Mansoura University, Mansoura, Egypt; 5 Animal and Human Health program, International Livestock Research Institute, Ouakam, Dakar, Senegal; 6 Department of Biochemistry and Molecular Biology, Institute of Biosecurity and Microbial Forensics, Oklahoma State University, Stillwater, Oklahoma, USA; 7 Department of Biochemistry and Molecular Biology, Oklahoma State University, Stillwater, Oklahoma, USA; University of Maryland School of Medicine, Baltimore, Maryland, USA

**Keywords:** *Salmonella enteritidis*, poultry, antimicrobial resistance, whole-genome sequencing

## Abstract

Whole-genome sequencing (WGS) was used to characterize four *Salmonella enterica* Enteritidis isolates from poultry (*n=2*) and human (*n=2*) from Ouagadougou, Burkina Faso. Antimicrobial resistance genes, chromosomal mutations, and mobile genetic elements were identified by analysis of WGS data using sequence homology.

## ANNOUNCEMENT

*Salmonella* strains were isolated from slaughtered chicken feces in four open markets of Ouagadougou, a capital city of Burkina Faso with nearly 3 million residents. The strain isolation using the pré-enrichment step in buffered peptone water and the enrichment step in Rappaport-Vassiliadis broth and identification was done according to the protocol described by the International Organization for Standardization (1). Bacterial isolates were confirmed using API 20 E (bioMérieux, Marcy-l'Etoile /France).

Whole-genome sequencing of isolated *Salmonella* strains was done at the United States Department of Agriculture, Agricultural Research Service (USDA-ARS), Athens, Georgia. Five milliliters of overnight cultures grown in Lysogeny Broth was subjected to genomic DNA extraction using the GenElute Bacterial Genomic DNA kit (Sigma-Aldrich, St. Louis, MO). Extracted gDNA was quantified using the Qubit double-stranded DNA (dsDNA) high-sensitivity (HS) assay kit according to the manufacturer’s instructions (Life Technologies, Inc., Carlsbad, CA). DNA libraries were prepared using the Nextera XT DNA library preparation kit and Nextera XT index primers (Illumina Inc., San Diego, CA). The library fragment size distribution was checked using Bioanalyzer 2100 with an Agilent HS DNA kit (Agilent Technologies, USA) and quantified using a Qubit DNA HS assay kit in a Qubit 2.0 Fluorometer (Life Technologies, Inc., Carlsbad, CA). The generated libraries were then sequenced using a MiSeq version 2 reagent kit. The paired-end read length of 2 × 250 bp was used for 500 cycles on the Illumina MiSeq platform. The quality metrics of the reads were performed by FastQC (http://www.bioinformatics.babraham.ac.uk/projects/fastqc/). All analyses were done with default program parameters unless otherwise noted. The sequence data were assembled using the A5-miseq assembler (2), and the genome sequence was annotated *via* the NCBI Prokaryotic Genome Annotation Pipeline (3). Genome size (Mb) is 4.75 for strain 61, 4.59 for strain 62; 4.73 for strain 28, and 4.73 for strain 29. N50 (bp) was 358,739 for strain 61, 424,693 for strain 62, 49,784 for strain 28, and 52,196 for strain 29. The No. of Contigs was 56 for strain 61, 51 for strain 62, 185 for strain 28, and 171 for strain 29. The GC content (%) was 52.2 for strain 61, 52.1 for strain 62, 52.2 for strain 28, and 52.2 for strain 29. The number of sequencing reads generated for each isolated was 1,150,754 for strain 28; 1,140,420 for strain 29; 2,469,466 for 65 strain 6; and 424,693 for strain 62.

Antibiotic resistance genes and chromosomal mutations were identified using ResFinder 3.2 (4). SeqSero 2 was used to determine the serotypes of salmonella strains from genome assembly data (5). MLST was identified using the MLST database from the Center of Genomic Epidemiology (6). The PlasmidFinder was used to detect plasmid from the strains (7). Plasmid replicon types, IncI1 and IncQ1, were identified (Table 1).

**TABLE 1:** Antibiotic resistance, virulence genes, and MLST type present in the genomes of *Salmonella enteritidis* isolates^*a*^

Sample ID	Sources	Year	Resistance genes	Phenotypic resistance	Plasmid replicon type	Virulence genes	MLST
S28	Human	2017	aac(6′)-Iaa, blaTEM-1B, sul2, dfrA7, qacE, tet(B), catA1, aph(6)-Id, aph(3")-Ib	AMPICI (AMP); AMPSUL (A/S2); MINOCY (MIN); PIPERA (PIP); TET; TICCLA (TIM2); TRISUL (SXT)	IncI1(Alpha); IncQ1	lpfC;lpfD; lpfB; lpfA;lpfE; cheD; rtxB; entD; upaH; tar/cheM; fimI	11
S29	Human	2017	aac(6′)-Iaa, blaTEM-1B, sul2, dfrA7, qacE, tet(B), catA1, aph(6)-Id, aph(3")-Ib	AMPICI (AMP); AMPSUL (A/S2); MINOCY (MIN); PIPERA (PIP); TET; TRISUL (SXT)	IncI1(Alpha); IncQ1	lpfC; lpfB; lpfE; lpfA; lpfD; cheD; tsr; rtxB; vfr; tar/cheM; upaH	11
S61	Poultry	2015	aac(6′)-Iaa, blaTEM-1B, sul2, dfrA7, qacE, tet(B), catA1, aph(6)-Id, aph(3")-Ib	AMP; AMPSUL (A/S2); MIN; PIP; TET; TICCLA(TIM2); TRISUL(SXT)	IncI1(Alpha); IncQ1	soPA; ssaV; sopB/sigD; ssaC; sseC; ssaN; sseJ; steC; ssaU; ssaQ; csgG; csgG; sifB; sspH2; pipB; sseF; ssaT; sifA; sspH1; ssaJ; csgD; ssaK; sseG; ssaR; ssaL; sseB; steA; sseD; csgA; csgB; sscA;sscB; csgF; ompA; csgE; sopD2; spiC/ssaB; sseE; ssaP; steB	11
S62	Poultry	2015	aac(6′)-Iaa, bla-TEM-1B, sul1, dfrA7, qacE, tet(B), catA1, aph(3″)-Ib. aph(6)-Id	AMP; AMPSUL (A/S2); MIN; PIP; TET; TICCLA(TIM2); TRISUL(SXT)	IncI1(Alpha); IncQ1	misL; mgtB; lpfC; lpfD; lpfB; mgtC; lpfA; lpfE	11

^*a*^ Ampicillin (AMP), ampicillin/sulbactam (AMPSUL), minocycline (MIN); piperacillin (PIP), tetracycline (TET) ticarcillin/clavulanic acid (TICCLA); trimethoprim/sulfamethoxazole (TRISUL).

Antimicrobial susceptibility testing was performed using microbroth dilution following the protocol from TREK Diagnostics, (ThemoFisher, 168 Third Avenue, Waltham, MA USA 02451). Resistance values were determined using the CLSI guidelines M100S22 and read after 18 hours at 37°C using breakpoints reported previously (8).

This report of MDR *Salmonella* Enteritidis from Burkina Faso highlights the role of poultry as a potential reservoir of antimicrobial resistance in *Salmonella* Enteritidis in Burkina Faso.

## Data Availability

This whole-genome shotgun project has been deposited at GenBank under BioProject numbers PRJNA679582 and PRJNA926313PRJNA926313. The accession numbers for the registered samples are JAEAHH000000000 for strain 61, JADZRU000000000 for strain 62, JAQSLQ000000000 for strain 28, and JAQSLP000000000 for strain 29. The Accession number for raw reads is SRR26425235 for strain 61, SRR26425234 for strain 62, SRR26412996 for strain 28, and SRR26412995 for strain 29. The versions described in this paper are the first versions.
